# Progress on the Mechanisms and Neuroprotective Benefits of Dexmedetomidine in Brain Diseases

**DOI:** 10.1002/brb3.70116

**Published:** 2024-10-31

**Authors:** Zhenxing Tao, Pengpeng Li, Xudong Zhao

**Affiliations:** ^1^ Wuxi Medical School Jiangnan University Wuxi China; ^2^ Department of Neurosurgery Jiangnan University Medical Center Wuxi China; ^3^ Wuxi Neurosurgical Institute Wuxi China

**Keywords:** dexmedetomidine, intracerebral hemorrhage, neuropathic pain, stroke, subarachnoid hemorrhage, traumatic brain injury

## Abstract

**Introduction:**

Dexmedetomidine, a highly specific α2 agonist, has been extensively utilized in clinical sedation and surgical anesthesia since its introduction in 2000 due to its excellent sympatholytic, sedative, and analgesic effects. This review aimed to identify new approaches for the treatment of patients with brain disorders by thoroughly describing the mechanism of action of dexmedetomidine and examining its neuroprotective effects from the standpoints of basic and clinical research.

**Methods:**

The PubMed and Web of Science databases were searched using the keywords dexmedetomidine and related brain diseases, although relevant articles from the last decade were included for detailed summarization and analysis.

**Results:**

Dexmedetomidine has shown strong neuroprotective effects, such as protection of the blood‐brain barrier, decreased neuronal death, maintained hemodynamic stability, and reduced postoperative agitation and cognitive dysfunction. Furthermore, dexmedetomidine has been shown to exert various neuroprotective effects, including anti‐inflammatory and antioxidative stress effects, modulation of autophagy, and reduction of apoptosis in cerebral diseases.

**Conclusions:**

Dexmedetomidine acts as a neuroprotective agent against brain diseases during all phases of treatment. However, clinical trials with larger sample sizes are required to optimize dosage and dosing strategies.

## Graphical abstract

Dexmedetomidine has demonstrated potent neuroprotective effects in a variety of brain disorders, and this may be achieved by inhibiting inflammatory responses, attenuating oxidative stress, reducing apoptosis and autophagy, and improving the immune microenvironment.

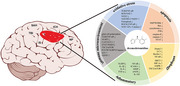

## 1 Introduction

Dexmedetomidine (DEX), an imidazole derivative, functions as a G protein‐coupled agonist of α2‐adrenergic receptors (α2‐ARs) and is predominantly processed by the liver and subsequently eliminated by the kidneys (Carnet Le Provost et al. 2024). DEX, a widely used benzodiazepine sedative, does not require the activation of the γ‐aminobutyric acid (GABA) system but directly stimulates α2‐AR excitation in the area of the Locus Coeruleus, which inhibits adenylate cyclase (AC) and reduces its activation, thereby exerting an effective central nervous system (CNS) depressant effect (Reid et al. 1997). Further, DEX accelerates the efflux and inhibits the influx of potassium from neurons, thereby reducing sympathetic excitation (Shirasaka, Kannan, and Takasaki 2007). It can also play an inhibitory role by lowering the level of norepinephrine to achieve anxiolytic, hypnotic, sedative, and analgesic effects, allowing patients to quickly enter non‐rapid eye movement sleep (McCarren et al. 2014). Moreover, DEX has shown a good synergistic effect when used in combination with other sedative and analgesic drugs, dramatically reducing the need for further sedative and analgesic drugs with fewer adverse effects and no respiratory depression, and effectively reducing the occurrence of postoperative cognitive dysfunction, delirium, and other complications. Because of these useful properties, it is widely used in clinics (Vallapu et al. 2018).

DEX is widely used for various types of surgical anesthesia, although studies have clinically verified that DEX offers superior sedation and analgesia compared with most other anesthetic drugs in the field of neurosurgery (D. Wang et al. 2024). DEX significantly improves sedation scores in patients under awake anesthesia, effectively maintains hemodynamic stability, enables the safe awakening of patients during surgery to maintain stable intracranial pressure, protects nerve cells, and reduces the incidence of postoperative cognitive dysfunction (M. Li et al. 2023). Relevant studies have further shown that DEX inhibits the inflammatory response, attenuates oxidative stress, reduces apoptosis and autophagy, modulates glial cell polarization, and improves the immune microenvironment (Y. Hu et al. 2022). As such, DEX may play a crucial protective role in the neurological recovery of patients with brain injuries. However, there are relatively few literature reviews concentrating on the process by which the neuroprotective advantages of DEX in brain diseases are achieved. Therefore, this review discusses the neuroprotective effects and related mechanisms of DEX in traumatic brain injury, cerebral hemorrhage, subarachnoid hemorrhage, stroke, neuropathic pain (NP), and other brain diseases in order to offer a more coherent theoretical framework and therapeutic approach for the use of DEX in brain diseases.

## 2 Traumatic Brain Injury

Traumatic brain injury (TBI) is characterized by brain damage and dysfunction caused by external forces (Arbour 2013). TBI causes synaptic dysfunction, protein aggregation, mitochondrial autophagy, and neuroinflammation through multiple mechanisms (X. Chen et al. 2022). Currently, most clinical interventions for TBI are based on surgery or symptomatic treatments such as reducing cerebral hypoxia, cerebral edema, and metabolic disorders (Khellaf, Khan, and Helmy 2019).

S. Y. Liu et al. (2024) showed that TBI patients with preoperative DEX had better Dysfunction Rating Scale (DRS) scores and were able to wean off mechanical ventilation earlier and maintain spontaneous respiration while undergoing mild sedation, which is very useful in agitated and uncooperative patients. Intraoperative infusion with DEX ensures better sedation, postoperative analgesia, and clinical recovery, reduces the frequency of delirium and the level of patient agitation, and suppresses the inflammatory response in traumatic brain injury patients with trauma debridement procedures and percutaneous tracheotomies, compared to conventional sedative medications such as midazolam (J. Gao et al. 2019; Soltani et al. 2021). In addition, X. Wang, Wang, and Cui (2022) found that DEX alone or in combination with butorphanol significantly improved pain and agitation scores and reduced the use of propofol and benzodiazepines in TBI patients. Patients treated with DEX had significantly lower scores on the Paroxysmal Sympathetic Hyperarousal Assessment Measure than those treated with standard sedation regimens of isoproterenol and midazolam, which could help to prevent and reduce the signs and symptoms (Hatfield et al. 2024).

**FIGURE 1:**
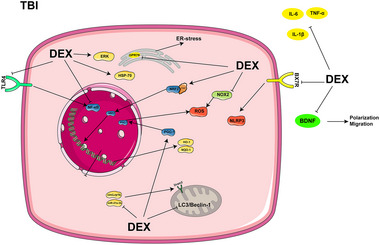
The mechanism of DEX in TBI.

Brain‐derived neurotrophic factor (BDNF), which is generally considered to exert beneficial effects, has a detrimental effect on TBI‐induced pain, although relevant basic research has found that DEX attenuates TBI‐induced chronic NP, possibly by decreasing the levels of BDNF in the cerebrospinal fluid (Jeon et al. 2023). BDNF also reduces microglial activation and migration, as well as monocyte‐derived macrophage infiltration in the hippocampal region, thereby inhibiting the expression of the inflammatory vesicle NOD‐like receptor pyrin domain‐containing protein 3 (NLRP3) and modulating the inflammatory vesicle NLRP3 via the purinergic 2×7 receptor (P2×7R), thereby attenuating the neurological damages (Karakaya et al. 2022; K. Sun et al. 2021). In addition, DEX regulates the autophagy process after TBI, reverses the up‐regulation of DNA damage‐regulated autophagy modulator 2 (Dram2) caused by circLrp1b and miR‐27a‐3p, and reduces the expression of autophagy‐related proteins, such as microtubule‐associated protein light chain 3 (LC3), Beclin‐1, and nuclear factor‐kappa B (NF‐κB), as well as the inflammatory factors interleukin‐1beta (IL‐1β), interleukin‐6 (IL‐6), and tumor necrosis factor‐α (TNF‐α), to inhibit neuroinflammation and to reduce neuronal death (Feng et al. 2021; H. Li et al. 2020). According to G. R. Huang and Hao (2021), DEX acts on the Toll‐like receptor 4 (TLR4)/NF‐κB pathway and the reactive oxygen species (ROS)/nuclear factor erythroid 2‐related factor 2 (Nrf2) signaling pathway to boost the levels of Nrf2 and the associated proteins heme oxygenase 1 (HO‐1) and NAD(P)H dehydrogenase, quinone 1 (NQO1). This successfully attenuates early neuronal damage and inhibits inflammatory responses. Furthermore, it enhances brain activity by preventing the overactivation of NADPH oxidase 2 (NOX2) (X. Chen et al. 2022; F. Li et al. 2019). DEX further reduces endoplasmic reticulum (ER) stress, activates the peroxisome proliferator‐activated receptor γ coactivator 1α (PGC‐1α) pathway, up‐regulates 70‐kDa heat shock proteins (HSP70) expression, and activates the extracellular signal‐regulated kinase 1/2 (ERK)‐related pathway following TBI, thereby inhibiting neuronal apoptosis, reducing axonal damage and synaptic degeneration, and promoting the recovery of motor function (F. Li et al. 2018; Schoeler et al. 2012; D. Sun et al. 2020; M. H. Zhang et al. 2018). Figure 1 represents an overview of the mechanism of action of DEX in TBI.

Overall, DEX exerts analgesic and sedative effects throughout the perioperative period in TBI patients. Further, it reduces the incidence of postoperative negative events, which may be achieved by inhibiting glial cell activation, suppressing autophagy, inhibiting inflammation and oxidative stress, and reducing neuronal apoptosis.

## 3 Intracerebral Hemorrhage

Intracerebral hemorrhage (ICH), defined as bleeding from broken blood vessels in the brain, causes brain tissue damage and neurological dysfunction and can result in both primary and secondary brain injury (Garg and Biller 2019). Associated primary brain injury is mainly attributed to the occupying effect of the hematoma, whereas extravasated blood components can induce inflammatory responses and oxidative stress pathways, causing secondary brain injury (Aronowski and Zhao 2011).

As an α2‐adrenoceptor agonist, DEX not only does not cause respiratory depression and affect hemodynamics when used intraoperatively but also acts synergistically with intravenous anesthesia. Consequently, it improves vital signs and reduces oxidative stress in patients with hypertensive cerebral hemorrhage and in those undergoing emergency craniotomy for cerebral hemorrhage (Shi et al. 2022). It also helps to reduce adverse events in patients with cerebral hemorrhage with indwelling tracheal catheters for 3 h postoperatively, improves postoperative sleep quality, exerts a positive effect on postoperative sedation, and is associated with a significantly better surgical prognosis than patients treated with midazolam (Q. Guo et al. 2021; J. Zhao and Zhou 2016).

**FIGURE 2:**
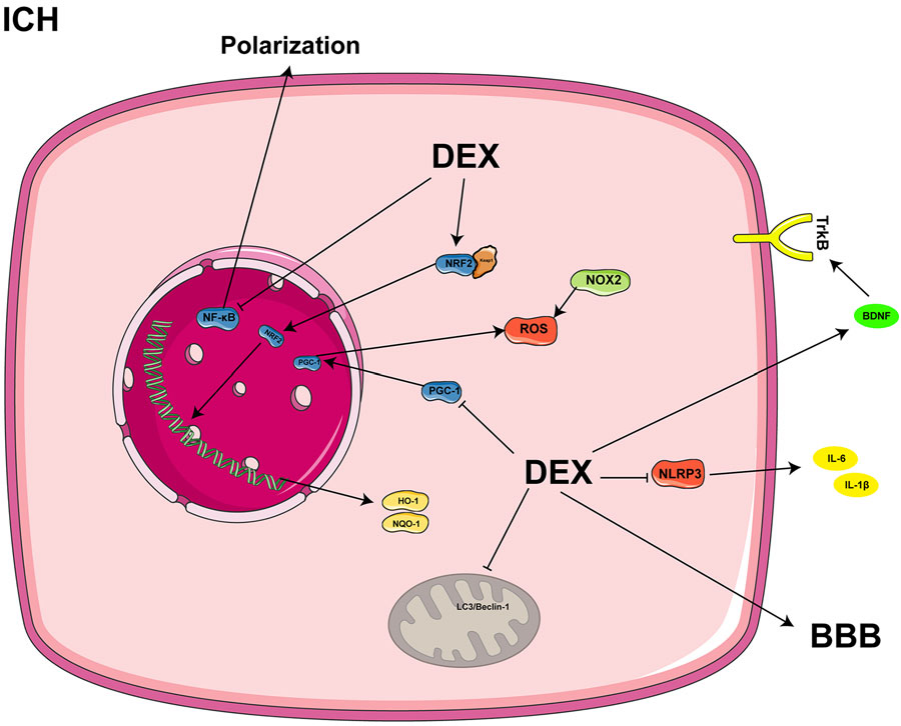
The mechanism of DEX in ICH.

In a recent study, DEX was found to attenuate cerebral hemorrhage‐induced short‐ and long‐term cognitive impairment, neurological dysfunction, and brain damage by modulating multiple pathways, including inhibiting the PGC‐1α pathway, restoring mitochondrial function, and enhancing BDNF and tropomyosin‐related kinase B (TrkB) expression (Huang and Jiang 2019; Hwang et al. 2013). Further, DEX exhibits potential anti‐inflammatory and antioxidant characteristics by inhibiting the activation of inflammasomes and lowering the levels of inflammatory cytokines such as IL‐6 and IL‐1β, and thus attenuating secondary brain damage and maintaining the stability of the blood‐brain barrier (H. L. Song and Zhang 2019). It further protects the blood‐brain barrier and ameliorates anxiety‐like behavior following cerebral hemorrhage by inhibiting key signaling pathways associated with the nuclear transcription factor NF‐κB, as well as modulating microglia/macrophage polarization (H. Guo et al. 2022). In addition, DEX inhibits neuronal apoptosis by activating the Nrf2/HO‐1/NQO1 signaling pathway, thus providing additional protection to brain tissue (Shao 2022). Nrf2 is a key regulatory component in ferroptosis, whereas DEX has been shown to be involved in regulating iron metabolism, amino acid metabolism, and lipid peroxidation through the modulation of Nrf2. This function reduces cellular damage caused by ferroptosis and further attenuates pathological changes after cerebral hemorrhage (M. J. Liu et al. 2022). Figure 2 shows the mechanism of action of DEX in ICH.

In patients with cerebral hemorrhage, DEX infusion attenuated hemorrhage‐induced oxidative stress, reduced postoperative complications, and enhanced sleep quality. This may be achieved through multiple pathways, including anti‐inflammatory and antioxidative stress, defense of the blood‐brain barrier, stopping neuronal death, and regulating ferroptosis.

## 4 Subarachnoid Hemorrhage

Subarachnoid hemorrhage (SAH), defined as bleeding in the subarachnoid space caused by the rupture of a cerebral aneurysm or vascular lesion, is one of the most common critical cerebrovascular diseases, with high mortality and disability rates (van Gijn, Kerr, and Rinkel 2007).

**FIGURE 3:**
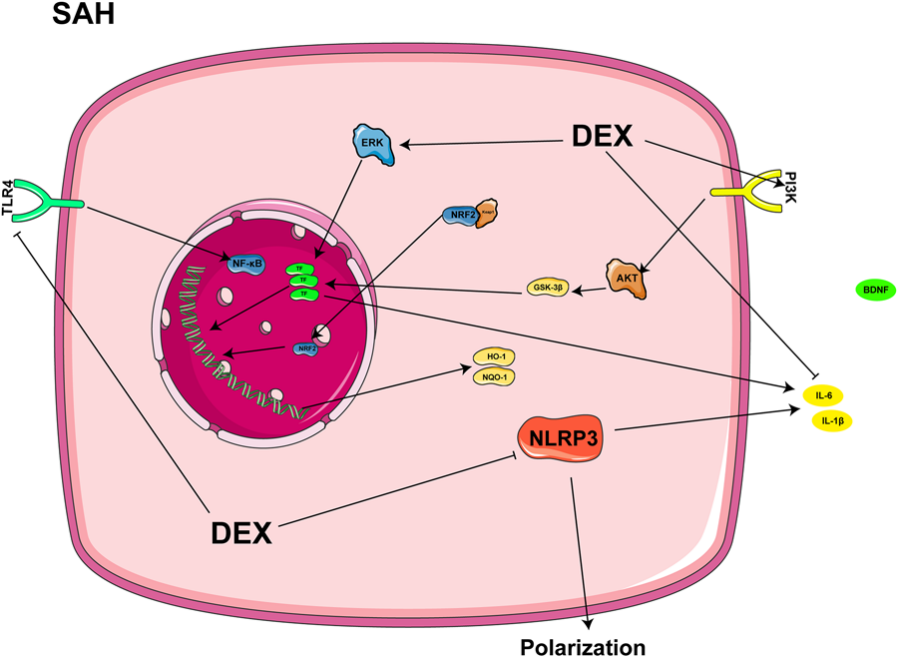
The mechanism of DEX in SAH.

DEX has multiple clinical benefits in patients with SAH demonstrated multiple clinical benefits (H. Liu, Busl, and Doré 2022). Research has shown that the administration of low‐dose DEX injection within 24 h of admission resulted in better neurological scores, especially compared to propofol, and reduced intraoperative adverse respiratory events and motor responses in patients with SAH (Okazaki et al. 2018). DEX was also superior to propofol when used as a sedative during cerebral angiography and was able to reduce total nimodipine consumption, maintain patient hemodynamic stability, and promote early recovery (Ren et al. 2019; Sriganesh et al. 2015). Recent studies have further shown that DEX at an initial loading dose of 0.5 µg/kg, followed by maintenance at a dose of 0.4 µg/kg/h, reduces the pentose phosphate pathway and nucleotide synthesis and exerts a beneficial impact on brain function in patients with SAH, without impairing static brain autoregulation (Kallioinen et al. 2020; Y. C. Li et al. 2022). Further, DEX reduces IL‐6 levels by activating the extracellular signal‐regulated kinase ERK‐associated pathway, contributing to the alleviation of oxidative stress and vasospasm following SAH (Y. Song et al. 2019; Wang, Han, and Zuo 2016). DEX has also shown potent anti‐inflammatory properties in SAH models, inhibiting the activation of the TLR4/NF‐κB signaling pathway and NLRP3 inflammatory vesicles, thereby reducing neutrophil infiltration and microglia activation, as well as increasing the release of proinflammatory cytokines and exerting neuroprotective effects (D. W. Han et al. 2022). DEX may help to reduce microglial pyroptosis in early brain injury (EBI) following subarachnoid hemorrhage by stimulating the phosphoinositide 3‐kinase (PI3K)/ protein kinase B (AKT)/ glycogen synthase kinase 3β (GSK3β) pathway (Wei et al. 2022). Figure 3 represents the mechanism of action of DEX in SAH process.

Overall, earlier use of DEX in patients with SAH is effective at improving neurological scores and reducing adverse respiratory events and motor responses. It further ensures safe sedation during cerebral angiography and promotes patient recovery. DEX protects brain function by modulating the associated metabolic pathways and attenuating oxidative stress, such as by inhibiting IL‐6 levels and vasospasm, and reducing neutrophil and microglia activation, the latter through the activation of specific signaling pathways such as ERK and PI3K/AKT/GSK3β, supporting the neuroprotective effects of DEX in patients with SAH.

## 5 Ischemic Stroke

Stroke, which is associated with high disability and mortality rates, is the primary cause of death in China and the second cause of death worldwide; consequently, it is considered one of the most important diseases that should not be neglected in clinics (Guzik and Bushnell 2017). Stroke is divided into hemorrhagic and ischemic stroke, of which ischemic stroke accounts for 70%. Stroke patients are often unable to live independently due to varying degrees of hemiparesis and speech impairment, which are life‐threatening and place a heavy load on patients and their families (Potter, Tannous, and Vahidy 2022).

In interventional stroke management, where patients often undergo procedures to ensure fine blood flow management, such as superficial temporal artery‐middle cerebral artery anastomosis and carotid endarterectomy, the use of DEX improves hemodynamic stability during the intraoperative, anesthesia awakening, and postoperative periods and is well tolerated by patients, achieving high satisfaction ratings (Kaku et al. 2018; Tsujikawa and Ikeshita 2019). Xue et al. (2021) found that DEX showed good results in pain and sedation management in the treatment of hypothermia due to neonatal hypoxic–ischemic encephalopathy, indicating its promising application in pediatric NP management.

MicroRNAs (miRNAs) are important regulators of gene expression and biological processes, particularly in the pathophysiology of ischemic stroke (Vasudeva and Munshi 2020). DEX, a potent neuroprotective agent, affects cerebral ischemia/reperfusion injury through multiple miRNA pathways. DEX upregulates miR‐148a‐3p, which consequently inhibits the signal transducer and activator of transcription (STAT)/Jumonji domain‐containing protein 3 (JMJD3) axis, prevents pyroptosis of astrocytes in the hippocampal region, and attenuates brain injury (Zhong et al. 2023). Further, DEX promotes the expression of other miRNAs, such as miR‐665, which reduces oxidative stress and inflammation and ameliorates ischemia‐reperfusion injury by inhibiting pathways such as Rho‐associated coiled‐coil containing protein kinase 2 (ROCK2)/NF‐κB (Q. Liu et al. 2022). DEX further protects neurons from hypoxic‐ischemic brain injury in newborn rats by upregulating miR‐10b‐5p and miR‐205‐5p, linked to the small nucleolar RNA host gene 16 (SHNG16), BDNF, and high mobility group box 1 (HMGB1) pathways (L. Wang et al. 2020; J. J. Yang et al. 2021). Furthermore, DEX regulates multiple miRNA target gene axes, including metastasis‐associated lung adenocarcinoma transcript 1 (MALAT1)/miR‐140‐5p/Nrf2, SNHG11/miR‐324‐3p/vascular endothelial growth factor A (VEGFA), miR‐337‐3p/Y box protein 1 (Ybx1)/lncRNA HOXA11‐AS, and circ‐CDR1as/miR‐28‐3p/tumor necrosis factor receptor‐associated factor‐3 (TRAF3). The regulation of these axes exerts a novel neuroprotective mechanism against ischemic stroke (Y. Chen, Fan, and Wu 2021; Qin and Xu 2024; J. Wang and Wang 2021; F. Yan et al. 2023). DEX further inhibits specific miRNAs, particularly miR‐323‐5p, miR‐199a, and miR‐29b, which ameliorate neuronal cell injury induced by cerebral ischemia‐reperfusion and reduce infarct size in animal model brains (Z. Huang et al. 2018; Seong et al. 2023; Y. Zhu et al. 2021). These studies indicate that DEX protects neuronal cells and reduces brain injury through the fine regulation of the miRNA network in multiple ways, offering a novel approach for treating ischemic stroke.

DEX has also shown positive effects in attenuating oxidative stress, inflammatory responses, and apoptosis. First, DEX inhibits oxidative stress and ferroptosis by activating the α2 adrenergic receptor, decreasing ROS levels, and enhancing related antioxidant capacities, such as increasing glutathione, catalase, and superoxide dismutase levels, while decreasing malondialdehyde and glutathione oxides (M. Hu et al. 2022; Liaquat et al. 2021). Further, it regulates the NOX2 signaling pathway by activating the kelch‐like ech‐associated protein 1 (Keap1)/Nrf2 signaling system, which exerts antioxidant activity and protects neuronal cells from hypoxia and reoxygenation‐induced cytotoxicity (X. Chen et al. 2021; G. J. Han et al. 2022). In terms of the inflammatory response, DEX inhibits a series of inflammatory cytokines and mediators, including IL‐1a, IL‐6, TNF‐α, NF‐κB, intercellular cell adhesion molecule‐1 (ICAM‐1), and matrix metalloproteinase (MMP), thereby lowering neuroinflammation and promoting neurological recovery (Peng et al. 2024). It also exerts neuroprotective effects through negative regulation of Janus kinase 2 (JAK2)/STAT3, HMGB1/TLR4/NF‐κB, and NF‐κB/cyclooxygenase‐2 (COX‐2) signaling pathways (H. Liu, Li, et al. 2022; Pan et al. 2016; Zhai et al. 2020). In terms of apoptosis, DEX was shown to attenuate brain damage following cerebral ischemia/reperfusion injury and cardiac arrest due to suffocation in rats by regulating the Bax mitochondrial cytochrome C‐caspase protease pathway, preventing c‐Jun N‐terminal kinase (JNK) phosphorylation, activating caspase‐8 and caspase‐3, and short‐term memory impairment (G. J. Wu et al. 2017; B. Zhao et al. 2021).

Autophagy, one of the key mechanisms underlying the protective effects of DEX, involves multiple pathways, including mitochondrial autophagy, peroxisomes, ER, and ribosomes (J. Wang et al. 2023). DEX further inhibits excessive autophagy in neurons and microglial cells through Sirtuin 3 (SIRT3)‐mediated activation of autophagy and up‐regulation of hypoxia‐inducible factor‐1α (HIF‐1α) and regulates mitochondrial dynamic homeostasis through 5' adenosine monophosphate‐activated protein kinase (AMPK) signaling and dynamin‐related protein 1 (Drp1) serine 637 phosphorylation to regulate mitochondrial dynamic homeostasis. These functions reduce the decline in neuronal density and axonal demyelination, thereby enhancing long‐term learning and cognitive performance (Fu et al. 2022; Luo et al. 2017; Xue et al. 2021; Zhou et al. 2021).

**FIGURE 4:**
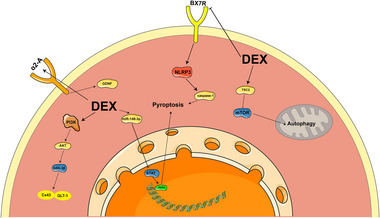
The mechanism of DEX in glial cells in I/R.

Astrocytes and microglia are the dominant non‐neuronal cells in the CNS, which not only play a key role in maintaining normal nervous system function but also play important regulatory roles in the inflammatory responses and disease processes. DEX upregulates astrocyte connexin 43 (Cx43) and glutamate transporter‐1 (GLT‐1) levels in astrocytes through activation of the PI3K‐Akt‐GSK‐3β pathway (Zheng et al. 2020), upregulates miR‐148a‐3p to deactivate the STAT/JMJD3 axis to inhibit astrocyte pyroptosis in the hippocampal region (Zhong et al. 2023), and further exerts neuroprotective effects by activating astrocyte autophagy by regulating the tuberous sclerosis complex 2 (TSC2)/mammalian target of rapamycin (mTOR) signaling pathway (C. Zhu et al. 2020). DEX further attenuates brain injury in hypoxic‐ischemic neonatal rats by inhibiting microglial cell death by blocking the P2×7R/NLRP3/Caspase‐1 pathway (K. Sun et al. 2021). Furthermore, DEX protects neurons by activating the α2A receptor to promote astrocyte function, including the release of growth factors such as glial cell line‐derived neurotrophic factor (GDNF) (M. Yan et al. 2011). Figure 4 shows the mechanism of DEX administration in glial cells.

**FIGURE 5:**
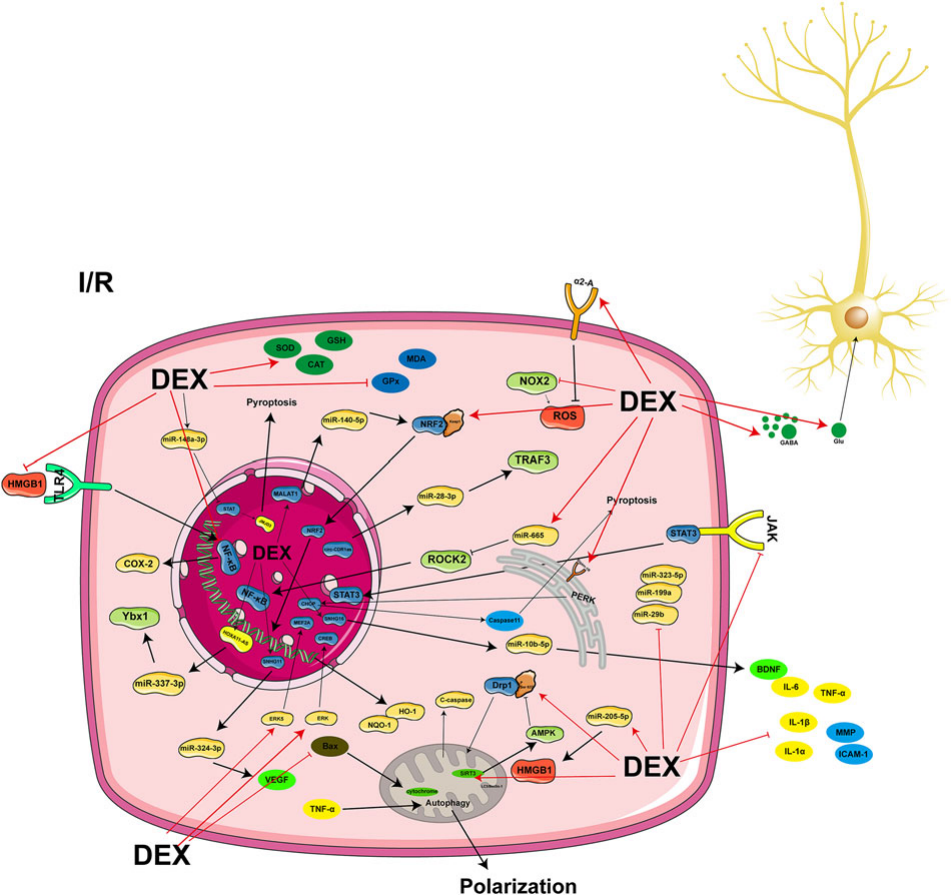
The mechanism of DEX in I/R.

Further, DEX ameliorates brain injury after ischemia/reperfusion through action on multiple pathways, including modulation of gene expression (e.g., SRY‐box transcription Factor 11(Sox11), neuroglobin (Ngb), and hypoxia‐inducible factor 1α (HIF‐1α), inhibition of harmful pathways (e.g., ferredoxin 1 (FDX1)‐mediated copper toxicity, sphingosine kinase type 1 (Sphk1)/ sphingosine‐1‐phosphate (S1P) signaling, NOX2‐mediated oxidative stress, and the JNK pathway, double‐strand RNA‐dependent protein kinase (PKR) ER‐resident kinase (PERK)‐C/EBP homologous protein (CHOP)‐Caspase‐11), and the activation of beneficial signals (e.g., ERK/cyclic adenosine monophosphate (cAMP)‐response element binding protein [CREB], PI3K/AKT, AMPK, ERK5/myocyte enhancer factor 2A [MEF2A], and Sigma‐1R), resulting in decreased neuronal damage, suppression of inflammatory responses, and a reduction of apoptosis (X. Chen et al. 2022; Cong et al. 2023; Y. Gao et al. 2020; Q. Guo et al. 2023; C. Liu et al. 2018; Teng et al. 2019; Y. P. Wang et al. 2023, 2019; Y. Zhu et al. 2019). In the MCAO model, DEX improves learning memory function, reduces brain injury, and promotes angiogenesis, possibly by up‐regulating the expression of HIF‐1α, VEGF, and BDNF (Kim et al. 2024). DEX can further reduce the levels of excitatory amino acids by increasing the uptake of glutamate, while increasing the concentration of the inhibitory amino acid GABA, thus attenuating the effects of brain damage (S. Lin et al. 2017). Figure 5 shows the mechanism of action of DEX in ischemic stroke.

In summary, DEX is a safe and effective treatment for patients with ischemic stroke, not only maintaining good hemodynamic stability but also reducing post‐ischemic agitation and pain. DEX further exerts neuroprotective effects through various mechanisms, including the regulation of miRNAs, reduction of oxidative stress, inflammatory response, apoptosis, glial cell polarization, and autophagy, indicating that it can exert neuroprotective effects on cerebral ischemic injury in various ways.

## 6 Neuropathic Pain

NP is defined as pain resulting from injury or illness of the nervous system (Bouhassira 2019). This pain, which is distinct from the normal pain caused by injurious stimuli, and results from abnormalities in the nervous system, such as nerve damage, inflammation, disease, or dysfunction (Finnerup, Kuner, and Jensen 2021). Multiple studies have indicated that DEX is effective at mitigating a range of pain conditions, including chronic pressure pain, surgical pain, and pain associated with diseases such as herpes zoster and diabetic neuropathy (Y. Zhao et al. 2020). DEX is further able to act as an adjuvant to peripheral nerve blocks, blocking the transmission of pain in the aδ and C fibers with mild anti‐injury effects, delivering patients with complicated regional pain syndromes rapid, long‐term pain relief (Sheikh and Baig 2023). In the treatment of acute herpes zoster, which causes intolerable pain and itching in patients owing to nerve invasion by the herpes virus, DEX reproducible paravertebral blockade therapy significantly reduces pain (F. Yang et al. 2022). A related study found that the intraoperative prophylactic intravenous infusion of DEX lowered the frequency of acute pain scores and persistent incisional pain following intracranial tumor resection. Additionally, in scalp nerve blocks performed postoperatively, the addition of DEX (1 µg/kg) to the local anesthetic bupivacaine extended the pain‐free time after craniotomy, and further reduced the need for opioids (McCullough et al. 2023; Zeng et al. 2024).

DEX is effective at alleviating pain symptoms, possibly through multiple molecular mechanisms, including blockade of pain signaling, anti‐injury sensation, anti‐inflammatory effects, neuroprotection, and improvement of nerve regeneration (Finnerup, Kuner, and Jensen 2021). DEX has further been shown to inhibit signaling pathways linked to NP, for example, by regulating the AMPK, p38 MAPK, NLRP3, Keap1‐Nrf2‐HO‐1, ERK1/2, and P2×7R pathways and modulating oxidative stress, inflammatory response, and apoptosis to attenuate NP in rats with modelled chronic contraction injury (T. Huang et al. 2021; J. P. Lin et al. 2018; Y. Liu et al. 2021; Mu et al. 2022; Shan et al. 2021; Yamakita et al. 2017). It further reduces the expression of neuronal nitric oxide synthase (nNOS) and certain inflammatory mediators in the spinal cord (TNF‐α, IL‐6, and IL‐1β), while increasing the expression of the anti‐inflammatory factor IL‐10, and further participates in the regulation of spinal ER phagocytosis through the inhibition of the stimulator of interferon genes (STING)/TANK binding kinase (TBK) signaling pathway, which is critical for the reduction of chronic NP (Y. Liu et al. 2022; Pang et al. 2023; X. Wang and Liu 2021). Further, DEX promotes the restoration of chloride cotransporter 2 (KCC2) function by affecting the BDNF/TrkB signaling pathway, which contributes to attenuating the effects of persistent postoperative pain (Dai et al. 2018). DEX also acts directly on primary sensory neurons to further reduce pain signaling by inhibiting transient receptor potential vanilloid 1 (TRPV1) channel expression (Lee et al. 2020). Simultaneously, it establishes a link between α2‐AR and Nav1.8 channels in these neurons through the Gi/o/AC/cAMP/protein kinase A (PKA) signaling pathway, thereby interfering with pain signaling, which contributes to the analgesic effect (Gu et al. 2015).

**FIGURE 6:**
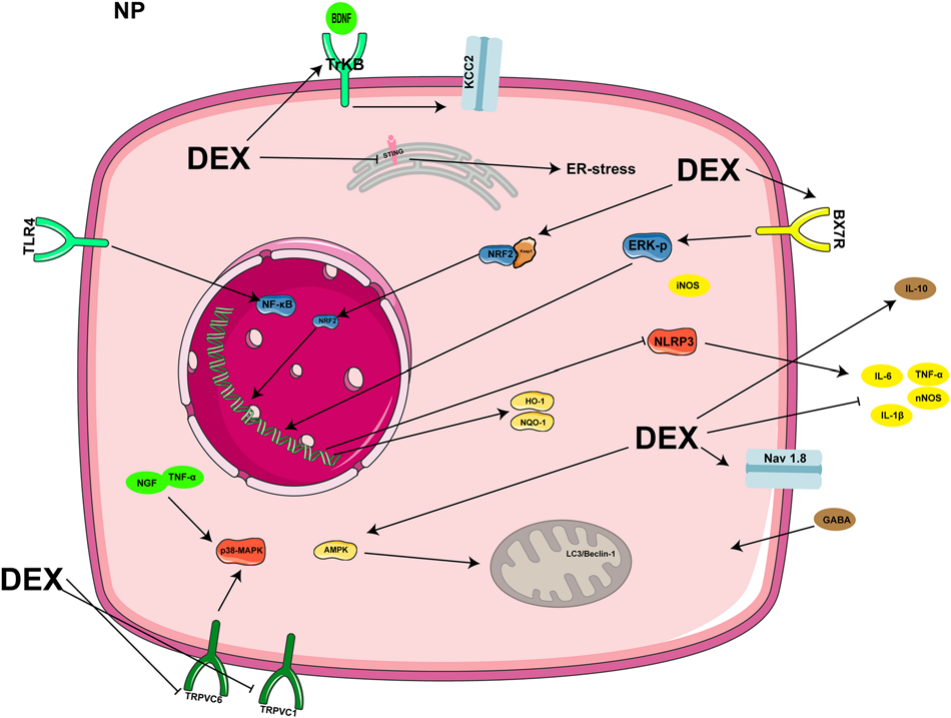
The mechanism of DEX in NP.

DEX inhibits the activity of cells in the dorsal horn of the spinal cord, such as astrocytes, microglia, and satellite neuroglia, reducing their activation and proliferation. It also controls the expression of the genes linked to them (e.g., nerve growth factor [NGF], transient receptor potential canonical subfamily member 6 [TRPC6], N‐Methyl‐D‐Aspartate receptor subunit 2B [NR2B], NF‐κB, and inducible nitric oxide synthase [iNOS]), resulting in improved neurological function and motor recovery (Liang et al. 2017; P. Liu et al. 2020; J. R. Wu et al. 2017; Xu, Yi, et al. 2022). In addition, DEX affects certain regions of the brain, including the dentate gyrus (DG) of the hippocampus, which may help to combat chronic pain‐induced depressive symptoms by promoting neurogenesis (Xu, Zhao, et al. 2022). Figure 6 shows the mechanism of action of DEX during NP.

Overall, DEX has been extensively shown to be effective at reducing chronic compressive nerve pain, neuropathy, herpes zoster neuralgia, and postcraniotomy pain. This action is achieved through multiple molecular mechanisms, such as blocking pain signaling, neuroprotection, and improving neurogenesis, anti‐injury sensation, and anti‐inflammatory effects.

## 7 Other Disease

The effects of cardiopulmonary bypass (CPB) on the brain mainly stem from its temporary substitution of blood circulation, which may lead to many pathophysiological alterations, including cerebral edema, oxidative stress, and acid‐base balance disorders, which may in turn affect the normal function of the brain (Jufar et al. 2023). Pre‐infusion of DEX partially attenuates CPB‐induced injury, by reducing the expression of p‐AKT and HO‐1, and attenuates cerebral nerve damage stemming from extracorporeal circulation (Xiong et al. 2022). Additionally, it has been shown to reduce cognitive impairment in rats that underwent CPB for heart surgery. This effect may be attributed to the inhibition of microglial activation, apoptosis, and inflammation in the DG regions of the prefrontal cortex and hippocampus (Z. Gao et al. 2021). The use of DEX in pediatric cardiac surgery further has a positive impact on neurodevelopment in children younger than 2 years, thus reducing the risk of postoperative neurodevelopmental problems (J. Huang et al. 2020). The neuroprotective effects of DEX are dose‐dependent and may attenuate brain damage after extracorporeal circulation by inhibiting the phosphorylation of JAK2 and STAT3 in the hippocampal regions (Y. Chen et al. 2017).

Sepsis has severe effects on the brain and can lead to a condition known as septic encephalopathy (SEP), which causes cognitive impairment, altered consciousness, brain edema, and neuronal damage (Z. Zhang et al. 2024). Studies in sepsis models have further shown that DEX can protect the brain, improve blood–brain barrier function, and reduce systemic/neurological inflammation and cognitive impairment (Tang, Zhong, and Wu 2024). These functions may be related to the modulation of the α2A adrenergic receptor and heat shock protein 90 (Hsp90)/AKT pathways (Mei, Li, and Zuo 2021; Yin et al. 2019). DEX has also been demonstrated to exert a protective effect on brain function and enhance the prognosis of sepsis by reducing glial cell pyroptosis, which safeguards neurons (Y. B. Sun et al. 2019). Additionally, owing to its anti‐inflammatory and anti‐apoptotic properties, it can counteract the neurodegenerative alterations and neuronal apoptosis induced by lipopolysaccharide (LPS) in the brains of septic mice (Ning et al. 2017).

In summary, extracorporeal circulation can cause multiple adverse effects on the brain, including cerebral edema and dysfunction; however, the preemptive use of DEX attenuates these injuries and protects neurons by inhibiting inflammation and apoptosis and improving blood‐brain barrier function. Similarly, DEX is beneficial in SEP, improving cognitive deficits and neurological function, with possible mechanisms involving the α2A‐adrenergic receptor, the Hsp90/AKT pathway, and anti‐inflammatory/anti‐apoptotic effects.

## 8 Conclusion

DEX has been shown to exert neuroprotective effects in various brain diseases. In traumatic brain injury surgery, DEX ensures better sedation and analgesia, reduces postoperative complications, inhibits immune cell activation and migration, regulates autophagy, and inhibits neuroinflammation and neuronal death. In the treatment of patients with cerebral hemorrhage, DEX improves vital signs, reduces postoperative adverse events, modulates hemorrhage‐induced cognitive impairment and neurological dysfunction, reduces the levels of inflammatory cytokines such as IL6, protects the blood‐brain barrier, and improves anxiety‐like behaviors through multiple pathways. DEX further reduces intraoperative adverse respiratory events, attenuates oxidative stress and vasospasm, and inhibits the release of inflammatory agents in patients with subarachnoid hemorrhage. In cerebral ischemia/reperfusion therapy, DEX regulates amino acid concentration via various pathways, including the modulation of various miRNAs, attenuation of oxidative stress, anti‐apoptotic autophagy, modulation of glial cell activation, and blockade of various harmful signaling pathways. It also effectively inhibits various types of pain, including post‐brain surgery pain, chronic compression neuralgia, herpes zoster, and other neuropathic pain. It can also reduce neuronal damage when cerebral blood flow is reduced during specific surgeries, such as cardiac surgery and extracorporeal circulation, and can reduce damage to brain tissues stemming from systemic diseases such as sepsis. Interestingly, in a recent meta‐analysis, Gatica et al. (2023) found that DEX was able to exert potent modulation of IL‐1β and IL‐6 production in the CNS and was able to reduce apoptosis and oxidative stress in vitro. In summary, DEX, a potent neuroprotective agent, may attenuate brain damage in surgical and pathological conditions, improve cognitive function, and provide neuroprotection in complex diseases such as sepsis through multiple mechanisms. These findings support the application of DEX in neurosurgical anesthesia and intensive care, particularly in situations where neuroprotective strategies need to be considered.

Despite its neuroprotective effects, DEX may trigger side effects or adverse reactions under certain circumstances. For example, it may cause severe hypertension at the onset of infusion in intravenous anesthesia, which is dangerous for patients with cerebral hemorrhage and TBI, a condition related to the activation of α2b receptors in a relatively short period of time (Y. Li et al. 2024). Additionally, in one observational cohort study, DEX use for MRI sedation in pediatric patients was found to cause hypotension in 5.7% of children. Rationally individualizing the use of this medication is important for reducing the incidence of cerebral hypoperfusion events (Cruz et al. 2024). In addition, potentially serious cardiovascular side effects should be considered. Both of the cases reported by Lichtsinn remind us that DEX may cause bradycardia or cardiac arrest in children (Lichtsinn, Sehgal, and Wilson 2023). Indeed, one study found that higher doses of DEX in patients with delirium may cause slowing of gastrointestinal motility and functional obstruction, which is surprising as no gastrointestinal reactions related to DEX have previously been reported with DEX (Alkaissi, Khudyakov, and Belligund 2021). In conclusion, when using DEX, it is important to personalize the medication and adjust the dosage according to the changes in the patient's physical condition to avoid heavy injuries to the patient due to side effects or adverse reactions. In addition, related studies have shown some shortcomings; for example, DEX has been shown to reduce postoperative adverse events in patients with cerebral hemorrhage, but the timing of patient visits may vary due to a variety of factors. Further, there are no studies comparing the changes in blood pressure after DEX infusion in patients with different time windows, which is important for the safety of DEX use. Despite the excellent sedative and analgesic effects of DEX in neonatal hypoxic‐ischemic encephalopathy (Acun et al. 2024), clinical data on the possible harmful or protective effects of DEX on children's neurological development and growth are lacking, as is how it affects cerebral blood flow and intracranial pressure in children, which needs to be elucidated by more clinical evidence and translational research. There are currently no standard DEX dosing regimens for chronic compressive pain or post‐brain surgery pain that are accurate in terms of dosage, start time, duration, and combination with other medications. In addition, the intensity of pain varies depending on the surgical approach, depth of the resection site, and functional areas of the brain affected; high‐quality prospective articles are required to illustrate this.

Although this paper reviews the various regulatory effects and mechanisms of DEX in neuroprotection, most have not yet been fully explained. The mechanism of action of DEX is often confined to in vitro or in vivo experiments, although few studies have verified its effects. The mechanisms of action of DEX are similar in various diseases; for example, all involve the inhibition of TBI, ICH oxidative stress, and inflammatory responses, with action through NRF2 and NF‐κB related pathways, indicating that DEX acts in the same way on specific cellular and molecular levels in the brain region. However, there are a lack of similar studies to illustrate this. In addition, the mechanism of DEX action in cerebral ischemia is complex, involving apoptosis, autophagy, oxidative stress, inflammatory response, and immune regulation. However, high‐quality articles validating these mechanisms are lacking. Some articles are too grouped, and their accuracy should also be considered. Finally, in severe systemic diseases such as sepsis, there is no clear indication of DEX dosage and duration of use, which is still in the experimental stage; as such, further clinical application is worth considering.

In conclusion, DEX is a promising neuroprotective agent that can act against brain diseases such as TBI, cerebral hemorrhage, subarachnoid hemorrhage, stroke, neuropathic pain, and so on, in all phases of preoperative, intraoperative, and postoperative treatment. It functions through mechanisms that regulate inflammation, oxidative stress, apoptosis, autophagy, glial cell polarization, and miRNA expression. Nevertheless, this study had some limitations. To overcome these shortcomings, future studies are needed to optimize the dosage and administration strategy, expand sample diversity, explore the mechanism of action in depth, and conduct more clinical trials to validate its safety and efficacy in real medical situations.

## Author Contributions

**Zhenxing Tao**: writing–original draft, conceptualization, methodology. **Pengpeng Li**: data curation. **Xudong Zhao**: writing–review and editing.

### Peer Review

The peer review history for this article is available at https://publons.com/publon/10.1002/brb3.70116.

## Data Availability

Data sharing not applicable to this article as no datasets were generated or analyzed during the current study.
